# Case report: KETOLAND the psychoeducation program for ketogenic diet

**DOI:** 10.3389/fpsyt.2023.1155717

**Published:** 2023-06-08

**Authors:** Martina Paola Zanaboni, Ludovica Pasca, Maria Angela Geraci, Costanza Varesio, Monica Guglielmetti, Anna Tagliabue, Serena Grumi, Valentina De Giorgis

**Affiliations:** ^1^Department of Child Neurology and Psychiatry, IRCCS Mondino Foundation, Pavia, Italy; ^2^Department of Brain and Behavioral Sciences, University of Pavia, Pavia, Italy; ^3^Research Center CBPT, University of Rome (LUMSA), Rome, Italy; ^4^Department of Public Health, Experimental and Forensic Medicine, Human Nutrition and Eating Disorder Research Center, University of Pavia, Pavia, Italy; ^5^Laboratory of Food Education and Sport Nutrition, Department of Public Health, Experimental and Forensic Medicine, University of Pavia, Pavia, Italy

**Keywords:** psychoeducation, Cognitive Behavioral Play Therapy, therapeutic storytelling, ketogenic diet therapy, glut1 deficiency syndrome (GLUT1DS), chronic disease

## Abstract

Glucose transporter type 1 deficiency syndrome (GLUT1DS) is a rare neurological disorder characterized by a wide spectrum of symptoms: epilepsy, movement disorders and neurocognitive impairment. The gold standard treatment for GLUT1DS are ketogenic dietary therapies (KDTs), specifically classical ketogenic diet (CKD). Despite the benefits, CKD often represents a challenge for patients and their families since meal preparation is extremely demanding and deviates a lot from normal diet. To assure an optimal compliance to CKD a psychological support for parents and patients with GLUT1DS is highly recommended. Specifically, a psychoeducational intervention that ameliorates the knowledge about the illness and its therapy improves treatment' s adherence and efficacy. The aim of this case report is to investigate the effectiveness of a psychoeducational program, partially implemented through telepsychology, based on the theoretical model of Cognitive Behavioral Play Therapy (CBPT) to support KDT knowledge and adherence in a patient with GLUT1DS who presented a worsening of her clinical picture due to a sparse knowledge of KDTs principles which determined a low adherence. Thus, with this case report we propose a model of intervention with psychoeducation in a patient with a complex chronic disease.

## 1. Introduction

**Figure 1:**
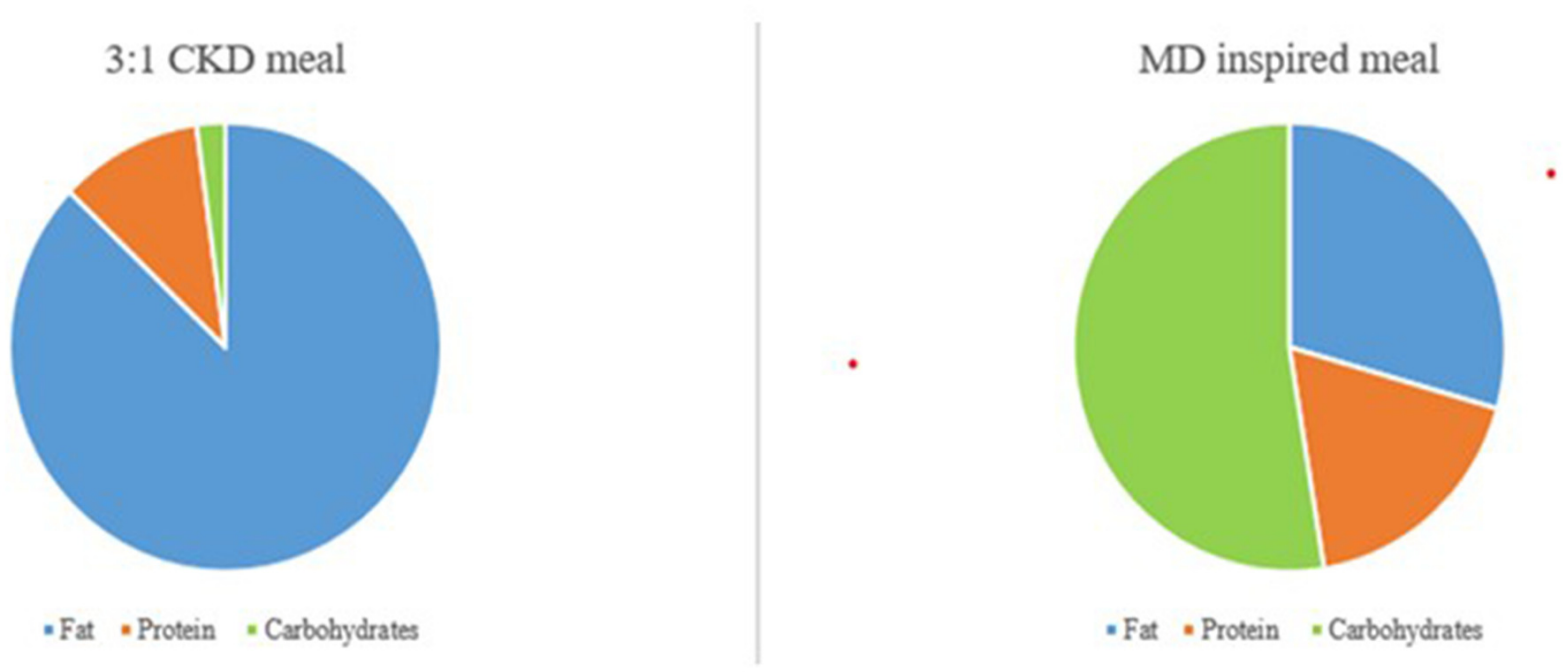
Graphical difference between macronutrients' composition of a 3:1 CKD meal and a MD inspired meal.

Glucose transporter type 1 deficiency syndrome (GLUT1DS) is a rare neurological disorder caused by impaired glucose transport across the blood-brain barrier, which results from haploinsufficiency of the *SLC2A1* gene (1). Affected patients classically present with infantile-onset epilepsy, impaired psychomotor development, intellectual disability complex movement disorders, and microcephaly, but several atypical variants of GLUT1DS have also been recognized (1). Interpersonal skills and adaptive social behavior usually represent the strengths of these patients (2). The gold standard treatment for GLUT1DS is Ketogenic dietary therapy (1). Ketogenic dietary therapies (KDTs) include 4 types of high-fat and low-carbohydrates ketogenic diets with published evidence supporting their use: classical ketogenic diet (CKD), the most restrictive type, medium-chain triglycerides ketogenic diet (MCT-KD), Modified Atkins Diet (MAD) and Low Glycemix Index Therapy (LGIT). Classic ketogenic diet is usually the first treatment choice for patients with GLUT1DS (3). It is a high-fat (80–90% of daily energy from fats), low-carbohydrate (about 3–7% of calories), adequate-protein dietary, that requires foods to be weighted at the nearest gram, to respect a precise ketogenic ratio (KR), defined as [lipids: (proteins + carbohydrates)]. The most commonly used are 3:1 and 4:1 classical KDTs (3), which are characterized by 3 and 4 grams of fats every 1 gram of carbohydrates plus proteins. Classic ketogenic diet mimics the metabolic state of fasting, providing ketones as an alternative fuel for the brain, thus restoring brain energy metabolism (3). They are extremely different from the “ketogenic” diets which are becoming popular for weight loss purposes. In fact, the latest ones are generally very restricted in calories (800–1,000 kcal/day),the so called “Very Low Calories Ketogenic Diets” (VLCKD). In contrast, classical KDTs are iso-caloric diets, low-moderate energy restriction is done only with overweight/obese patients that need weight loss. If adequately followed, KDTs, specifically CKD, usually permit an optimal seizure control, a discrete control of the movement disorder, as well as an improvement of cognitive abilities (1). Despite the outstanding benefits, KDTs often represent a challenge for patients and their families because it might be restrictive, requiring a demanding meal preparation (4). In order to show the differences in the preparation and distribution of macronutrients between a classical ketogenic meal vs. a meal inspired by Mediterranean Diet (MD) principles we provide an example in Figure 1.

The carbohydrate restriction, necessary in KDTs and maximum in the CKD, is one of the challenges that make the diet adherence difficult due to the limited food options and decreased palatability (4). Nevertheless, the dieticians might provide solutions to solve the problem of palatability with an individualized ketogenic dietary therapy plan. Moreover, this therapy requires constant nutritional monitoring over time both to ensure its effectiveness and to reduce the likelihood of short- and long-term adverse effects (3). Furthermore, caregivers need to understand complex instructions about diet preparation including strict weighing of foods. These therapies may be difficult for children of parents with low levels of literacy and poor socioeconomic status and access to resources (5). Taking into consideration the reported issues, the need for psychological support for parents and patients with GLUT1DS is highly recommended to assure an optimal compliance to KDT. The importance of psychological aspects of people with chronic illnesses has been pointed out by the Psychological Task Force of the International League Against Epilepsy (6) which has drafted evidence-based clinical recommendations regarding psychological treatments for people with epilepsy. These recommendations are suitable and applicable to people with GLUT1DS and focus on three issues: psychological screening, psychoeducational interventions (PE), and treatment adherence. In particular, in patients with GLUT1DS, non-adherence to KDT is of the utmost importance since inadequate compliance could cause failure of seizure control, a decrease of cognitive performances, and an increase of movement disorder. Educational programs addressed to patients and families with chronic diseases have been developed and implemented in past years (7, 8), yet target programs addressed to patients and families undergoing KDTs have not yet been developed. In this case report, we present our PE program named “KETOLAND,” an intervention aimed to implement knowledge and education regarding the disease, treatment, comorbid conditions, and challenges in following the KDTs. The program consists of two independent yet matched sections: one meant for the children affected by GLUT1DS and one for the parents. Both interventions face the same topics using several techniques and tools. Different stages of the intervention will be proposed: questions and answers, brainstorming, slides, videos, and therapeutic storytelling in order to develop more adaptive coping strategies and to face the emotional aspects. The authors believe that the possibility of implementing the program with both the caregivers and the patient is a crucial point. Specifically, a PE intervention has been scheduled to help both the parents and the patient to understand the disease, the needed treatment, and the physical and mental health consequences.

Regarding the methodology, the PE is a Cognitive Behavioral Therapy (CBT) technique which represents a systematic and structured approach aimed at ameliorating the knowledge about the illness and its treatment, integrating emotional and motivational aspects, to enable patients to cope with the illness and to improve treatment adherence and efficacy (9). The International League Against Epilepsy recommendations specify that for pediatric patients PE should be adapted taking into account the developmental ability of the patient, recommending the use of play as a therapeutic tool. Cognitive Behavioral Play Therapy (CBPT) is an appropriate supplement for the development of CBT and Play Therapy (PT) (10, 11). CBPT is accessible to young children through the integration of cognitive behavioral interventions with play. Taking into consideration that young children and children with intellectual disability are in a pre-operational stage therefore they are self-centered and concrete, CBT must be modified so that it does not rely on sophisticated language and the use of logic (10). The difference between CBT and CBPT is that interventions are adapted for the developmental level of the child. Moreover, this approach is tailored to the developmental level and needs of the child that assures indirect communication through play. Throughout CBPT the child can have access to the psychoeducational and therapeutic effects of the approach (11). One of the CBPT tools employed is therapeutic storytelling, which enables children to promote more adaptive thoughts, emotions, and problem-solving skills (12). Several studies reported that patients with chronic conditions encounter difficulties associated with disease-related stressors and decreased quality of life (13–15). During the COVID pandemic, due to limitations related to social distance, telemental health and remote monitoring (16, 17) became the standard of care. The American Psychological Association defines telepsychology as the “*provision of behavioral and/or mental health care services using technological modalities*” (18). Telepsychology turned out to be helpful to reduce barriers of time, distance, stigma, and costs representing a sustainable method of health care delivery. The aim of this case report is to investigate the effectiveness of a psychoeducational program partially implemented through telepsychology, based on CBPT to support KDT knowledge and adherence.

## 2. Case presentation

### 2.1. Demographic data and reason for referral

The clinical case reported regards a patient with GLUT1DS, first born, who lives in a family with a low socio-economic status. She had mild intellectual disability with generalized academic and attentional difficulties, and poorly controlled absence seizures associated with multiple generalized epileptiform discharges in treatment with Valproic Acid, Ethosuximide, Clobazam and Topiramate. She received the genetic diagnosis of GLUT1DS at the age of 11 years and at the same time KDT was started. At the time of the assessment, the patient was a 12 years old girl and was attending the 2nd grade of secondary school. Reasons for psychological referral were attention and school difficulties, poor relationships with peers, irritability and poor CKD adherence since introduction. Scarce CKD compliance was confirmed by very low blood ketone bodies levels (<1 mmol/L compared to the therapeutic ones 2–5 mmol/L).

### 2.2. Initial assessment and KETOLAND program

The adopted psychoeducational program was named “Ketoland” and was entirely held at the IRCCS Mondino Foundation. Written and informed parental consent was obtained for participation in the research. The treatment lasted 3 months and consisted of 1 once-a-week session, held by a multidisciplinary team composed of a pediatric neurologist, a psychotherapist, and a dietician. Both face to face sessions and telepsychology were implemented. To investigate the effectiveness of the PE intervention the following measures have been analyzed before and after the KETOLAND program: ketonemia, EEG, dietary treatment knowledge, and CKD adherence using a 7-day food diary and semi-structured psychological interviews. The main topics approached with the patient during the individual interviews were awareness about her medical situation and CKD adherence. The girl showed poor knowledge about her syndrome and treatments, specifically she did not know the reason why she had to follow the CKD.

The parents reported two main areas of concern, regarding relationships with peers and poor dietary adherence. The patient often appears nervous due to the teases held by her classmates. The teases concerned her physical appearance, clumsiness, and her different dietary habits. Connected to this first problematic situation, the parents reported that when the patient is nervous, she secretly eats foods not allowed in her dietary regimen. Taking into consideration the neurocognitive and emotional difficulties observed, a psychological assessment to better understand the child's functional profile and to design a supportive intervention was scheduled.

For the case conceptualization, the psychotherapist used the ABCE model (see Table 1), which is a cognitive-behavioral technique (19). In A-column, the *antecedents or discriminative stimuli* (triggers, signals) capable of triggering C (problematic responses) are reported. In column B, there are the *beliefs* which are the subjective evaluations of A based on own aims and beliefs. Column C shows the *coping responses* (behaviors, emotions, mental activities, and physiological reactions); coping responses can be of three types: a) avoidance b) surrender c) overcompensation. In column E, *the effects* and functions of 'C' on the maintenance of the problem are shown; the effects have the function of avoiding something aversive ($-), and/or to obtain conditions that are desirable for the individual (S+), and/or lead to undesirable effects (S-).

**Table 1:** Case conceptualization ABCE model (19), interview with the patient.

**A**	**B**	**C**	**E**
At home when she is not observed by her parents When she thinks about the mockery	“This is a good moment to step out of the line” “Why I can't eat like others? What will be the consequence?” “I don't want to be the strange one”	Problematic coping When she doesn't' follow her dietary regimen Physiological responses I feel a hole in my stomach Emotion Angry Mental activities Rumination “I am not like others”	S-: Fear of being sick. feel of guilty S+: pleasure and gratification. I like junk food, when I eat it I feel happy. $- Removal of pressing desire

To collect data regarding CKD-related knowledge and behavior, we administered three semi-structured interviews (see Supplementary material) aimed to investigate:

Knowledge about GLUT1DS.
Knowledge about CKD.
Adherence to CKD.

The interview with the patient revealed social desirability, she denied the episodes in which she didn't follow the diet.

The interviews with the parents were held individually with the parents. They showed adequate knowledge regarding GLUT1DS, while the patient does not know the causes of her disease. Concerning knowledge about KDT, the father had mistaken beliefs concerning the purchase of food and poor knowledge regarding where and how to find information about the diet (e.g., new recipes), and regarding foods that need to be reduced during C. The father also stated that he does not know the reason why it is necessary to follow the dietary treatment knowledge, and CKD adherence using a 7-day food diary and semi-structured psychological interviews. The interview with the mother confirmed the insufficient information regarding how to find information about the diet and new recipes. Concerning CKD adherence, the interviews with the parents revealed that the low adherence is due to difficulties in understanding how to manage the CKD. The family reported that they used to search new recipes on websites, some of them dedicated to weight loss. Moreover, when they wanted to add new foods, the choice was based on foods with zero added-sugar or “light.” This decision, however, appeared incorrect because although some foods are labeled as “zero fat” they may contain carbohydrates. From the analysis of the interviews, we outlined an insufficient knowledge of KDT which at least in part explained the poor adherence to the diet.

### 2.3. Aim of the program

We decided to prioritize the problem of adherence to CKD, with the aim of increasing knowledge about the disease and the treatment throughout PE intervention. In a nutshell, the hypothesis has been that the improvement of knowledge on the disease and the CKD, through a targeted PE program, could have led to a better treatment adherence.

### 2.4. Structure of KETOLAND program

The main aims of the PE intervention were increasing knowledge regarding the disease and improving self-management practices and CKD compliance. For the child therapeutic storytelling was used, for the caregivers different tools such as videos, brainstorming, questions and answers form were used. The therapeutic storytelling was applied through the creation of a booklet entitled “*Ketolandia: in search of the ketone door*.” This booklet explained the story of a little girl diagnosed with GLUT1DS undergoing CKD. The therapeutic storytelling stages included the reading of the book, divided into different phases to improve understanding of the contents (20). By handling different material and tools an emotional and practical support was assured, by providing a dedicated space to manage complex emotions and difficulties in the management of disease.

The sessions with the family have been conducted by a multidisciplinary team composed of a psychotherapist, a pediatric neurologist, and a dietician. Three modules have been designed for intervention (see Table 2). Both with the patient and the family we shared the same notions achieving the same result through different but essential and complementary paths. The patient and the family participated in distinct meetings.

**Table 2:** Ketoland program: form and contents of the intervention of PE in Telepsychology.

**Domain**	**Contents**
Form 1 Knowledge regarding GLUT1DS	GLUT1DS symptoms etiology diagnosis
Form 2 Treatments	Treatment with KDT Treatment with ASMs Psychological treatment Neuropsychological treatment
Form 3 Difficulties	Everyday life and KDT Food choice Go to grocery store KDT and social life KDT and school
Follow up	Food diary

## 3. Results

Ten meetings with the family and 10 meetings with the child were scheduled with a weekly frequency. The medium duration of the meetings was equal to 45 min. The physician and the psychologist were present at all meetings, the dietician was present only during meetings dedicated to dietary topics. To investigate the effectiveness of the PE intervention the following measures have been analyzed: ketonemia, EEG, GLUT1DS, dietary treatment knowledge, and CKD adherence using a 7-day food diary and semi-structured psychological interviews.

**Figure 2:**
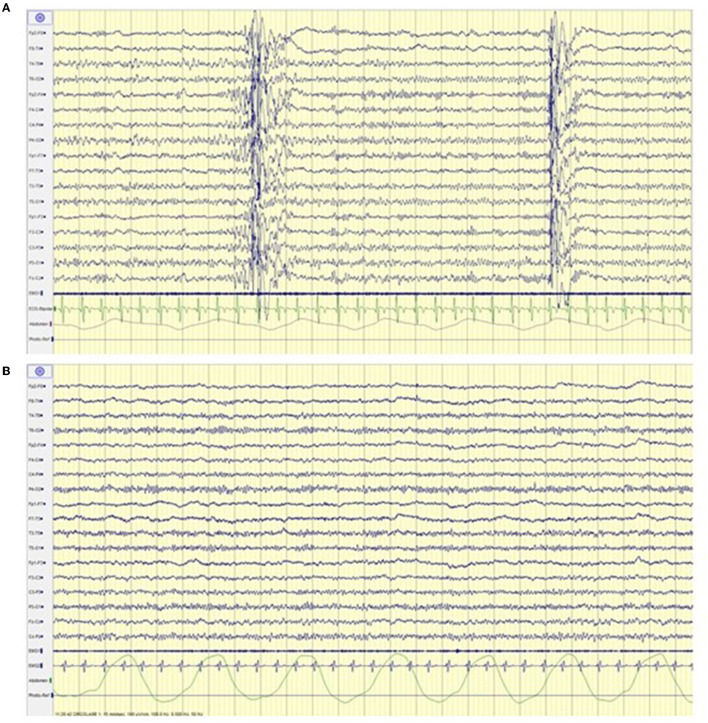
Electroencephalogram before **(A)** and after **(B)** ketoland intervention. **(A)** Generalized spike and polispikes and wave discharges. **(B)** Normal background activity without epileptiform abnormalities.

An increase in the ketonemia levels (score from <0.5 mmol/L during the first assessment to 2.3 mmol/L at the follow up) and a significant electroclinical improvement in terms of both seizure freedom and normalization of the electroencephalogram were registered (see Figure 2) that led to the initiation of the reduction of antiseizure medications (ASMs).

The psychological interviews with the family and the patient revealed an improvement of knowledge regarding CKD: foods that need to be reduced, the importance of adhering to nutritional prescriptions, and the identification of the KETO team as a point of reference in case of doubts and needs. Consequently, the improvement in CKD knowledge led to a better dietary adherence.

We report excerpts of the stages of therapeutic storytelling with G., during the “Feel it!” stage we asked “*How did you think that the* main *character feels? Talk about it. Create a diary entry of a character writing about her feelings*.” The patient wrote: “*Dear diary, today I read a story about ketones and a girl. I liked it so much. This girl's name was Luna, and she had the same problem as me. She told how her life went. I felt happy because she had the same problem as me*. Now, I k*now that I'm not the only one with this problem. Now I feel very well* “. During the “Own” stage we questioned her:”*Does what you read remind you of anything from your past? Tell me. How did you deal with these situations*?. The girl answered: “*I don't like to remember that I ate sugar, because it hurts my body. The thought makes me feel bad because I feel a little angry and a little sad. I ate sugar because I didn't know. Now I wouldn't take candy because I know it would hurt myself. I'm happy when I go to the hospital, because I see my doctors. I get blood tests, I get the octopus helmet, and I sleep. They are nice, they tell me what I have to eat and they measure my tummy. Then I'm an astronaut, they put a big thing on me to breathe, and I have to breathe. I do some exercises to train my intelligence and my attention*.”

## 4. Discussion

We reported the application of the KETOLAND program to a patient with GLUT1DS and her family, in order to improve the adherence to the very strict dietary regimen she had to follow. The low adherence to the CKD caused a worsening of the clinical condition. Often, despite the well-known benefit of available treatment, patients with a chronic disease, especially at some ages, struggle to adhere to medical indications. And the burden of KDTs in general, specifically CKD, for patients and their families is well recognized in clinical practice and in literature. Varesio et al. (21) investigated the presence of emotional and behavioral difficulties in a cohort of 9 pediatric patients with GLUT1DS on a stable classical KD treatment, revealing the presence of social problems related to the dependence on the caregiver due to the strict dietary regimen. To increase the adherence to treatment of the patient herein described, a psychological assessment and a specific PE program tailored to CKD were proposed.

Throughout the psychological interviews, we observed that the family showed a poor knowledge regarding the dietary therapy that led to a low adherence to treatment indications. The poor knowledge and adherence to KDT partly derived from poor cognitive competence of the family. A study by Govil et al. (22) investigated possible factors associated with low treatment adherence in patients with epilepsy, showing that patients with a low cultural and educational level might more commonly find difficulties in following medical prescriptions. In this specific case, a low cultural and educational level led to a poor understanding of both the pathology and treatments, generating dysfunctional beliefs and behaviors that caused poor adherence. To improve dietary knowledge and adherence we developed a PE program tailored to the specific genetic disorder and CKD. Even if PE interventions focused on CKD have not been standardized yet, different studies showed the efficacy of PE programs to increase knowledge and adherence to treatment in patients with chronic diseases (23, 24). Throughout the interviews and the targeted PE stages, we observed an improvement of awareness and adherence of both the patient and the family to CKD. The therapeutic storytelling was useful to aid the patient to express her emotions and feelings, promoting more adaptive behaviors and emotional responses. The proposed story reflected the child's identity and daily life, and the utilization of an easy language ensured a deep understanding, promoting more adaptive coping skills (11). Furthermore, our program showed that the improvement of CKD adherence brought an overall clinical improvement in parallel with a significant EEG amelioration, leading to discontinuation of ASMs and thus furtherly predisposing to a neuropsychological improvement.

Analyzing the strengths of the programme, the presence of a multidisciplinary team was crucial to ensuring accurate answers to medical, nutritional and psychological questions. In addition, the possibility of using telepsychology overcoming the distance and the costs represented a sustainable method of health care delivery with advantages both for the clinicians and the family. Indeed, the parents were more at ease and in a favorable position being at home, perceiving this intervention as a support in their daily life. The implementation of psychological interviews focus on diet related variables identifying the needs of the patient and her family and the variables which hinder the adherence to CKD. Considering avenues for future research, the creation of standardized tools to detect CKD knowledge and adherence would be useful for clinical purposes. Moreover, a future direction will be the proposal of the same program to other subjects with GLUT1DS or drug resistant epilepsy of other etiology and their families to improve disease knowledge and therapy compliance. The present study has some limitations since this psychoeducational method was applied to only a single patient, thus the results cannot be generalized; moreover, the follow-up provided is just in the medium term and the outcome of the intervention should deserve a re-evaluation in the long term.

This work emphasizes the importance of identifying factors that hinder adherence to treatment to promote psycho-education programs aimed to increase not only treatment compliance but also the patients' and families' general well-being and quality of life.

## Data availability statement

The raw data supporting the conclusions of this article will be made available by the authors, without undue reservation.

## Ethics statement

The studies involving human participants were reviewed and approved by Ethical Committee of the IRCCS Policlinico San Matteo Pavia P-20190033749. Written informed consent to participate in this study was provided by the participants' legal guardian/next of kin. Written informed consent was obtained from the individual(s) for the publication of any potentially identifiable images or data included in this article.

## Author contributions

MZ, LP, VD, MGe, and MGu contributed to the conception, design of the study, and wrote the manuscript. All authors contributed to manuscript revision, read, and approved the submitted version.
