# Supplementary material for: Case report: KETOLAND the psychoeducation program for ketogenic diet
Source: Front Psychiatry. 2023 Jun 8;14:1155717. doi: 10.3389/fpsyt.2023.1155717 (PMC10285047; doi:10.3389/fpsyt.2023.1155717)
Supplement: Supplementary file 1 [file Data_Sheet_1.docx]

***Supplementary Material***

**Martina Paola Zanaboni^1,+^, Ludovica Pasca^1,2,+*^, Maria A. Geraci^3^, Costanza Varesio^1,2,+^, Monica Guglielmetti^4,5^, Anna Tagliabue^4^, Serena Grumi^1^, Valentina De Giorgis^1,2,+^**

^1^ Department of Child Neurology and Psychiatry, IRCCS Mondino Foundation, Pavia, Italy

^2^ Department of Brain and Behavioral Sciences, University of Pavia, Pavia, Italy

^3^ Research Center CBPT, Rome, Italy, University of Rome (LUMSA), Italy

^4^ Department of Public Health, Experimental and Forensic Medicine, Human Nutrition and Eating Disorder Research Center, University of Pavia, Pavia, Italy

^5^ Laboratory of Food Education and Sport Nutrition, Department of Public Health, Experimental and Forensic Medicine, University of Pavia, Pavia, Italy

^+^Member of European Reference Network for Rare and Complex Epilepsies, EpiCARE, Italy

*** Correspondence:**Ludovica Pasca
[ludovica.pasca@mondino.it](mailto:ludovica.pasca@mondino.it)

Case report: KETOLAND the Psychoeducation program for Ketogenic diet

**Semi-structured interviews addressed to the family and the patient**

**1) Knowledge about GLUT1DS**

What is GLUT1DS?

Which are the main symptoms associated with GLUT1DS?

Which are the causes of GLUT1DS?

Which is the specific therapy for GLUT1DS?

**2) Knowledge about Classical ketogenic diet?**

What is the classical ketogenic diet (CKD)?

Which are the benefits of CKD?

Why is it necessary to follow the ketogenic diet strictly?

Who manages the prescriptions of CKD?

Which of the following foods are reduced when following the CKD?

Which food characteristics must have the food that you buy?

What you have to do when you prepare a meal?

If you have any doubts or uncertainties about a food or recipe, where can you find information about the CKD?

What do you should do when you want to include a new recipe or product in your CKD?

**3) Classical ketogenic diet adherence**

Do you think that following the CKD is easy?

In what situations is mostly difficult to follow the CKD?

Do you only prepare/ eat products included in your diet?

Do you weigh all the food?

Do you regularly fill the food diary?

Do you read the labels of all products you purchase?

Since you started the CKD have all the medicines used been replaced with sugar-free ones?

When you add a new recipe, do you contact the KETO team?
